# Inpatient Treatment for the Middle-aged and Elderly in Central China

**DOI:** 10.3389/fpubh.2017.00007

**Published:** 2017-02-02

**Authors:** Yan Jiang, Yu Wang, Yang Li, Yuming Zhang, Yinjun Zhao, Xiaojun Wang, Chi Ma, Shuangge Ma

**Affiliations:** ^1^Center for Applied Statistics, School of Statistics, Renmin University of China, Beijing, China; ^2^School of Public Health, Yale University, New Haven, CT, USA; ^3^Ideological and Political Education Center, Beijing Institute of Petrochemical Technology, Beijing, China

**Keywords:** inpatient treatment, middle-aged and elderly, characteristics, prevalence, cost, China

## Abstract

**Background:**

Compared to outpatient treatment and self-treatment, inpatient treatment corresponds to more severe illness and poses more serious health and financial burden to patients. The goal of this study is to provide an updated and comprehensive description of the prevalence, characteristics, and cost of inpatient treatment for the middle-aged and elderly in Central China, which is highly populated, less-developed, and agriculture-dominating.

**Methods:**

A survey was conducted in August 2013 in the Henan province. Data on 1,464 subjects were collected.

**Results:**

Among the surveyed subjects, 582 had at least one episode of inpatient treatment. Subjects with different inpatient treatment status differ in the distributions of age, education, occupation, area, health insurance coverage, physical condition, and presence of chronic disease. The surveyed subjects had up to six inpatient treatments within 12 months. Different episodes have different characteristics. Age and the presence of chronic disease are significantly associated with the number of inpatient treatments. The utilization of grade III hospital for inpatient treatment is associated with gender, marital status, and per capita income. The total and out-of-pocket costs are associated with education, utilization of type III hospital, and insurance utilization.

**Conclusion:**

This study has provided a comprehensive description of inpatient treatment for Central China, an area with low developmental and economic status. The observations may assist improving health conditions and disease treatment for this less-advantaged area.

## Introduction

The development of China’s healthcare system falls far behind its economic achievements (1). A system-wide reform took place in 2009, with the goal of improving quality and accessibility of healthcare and reducing cost (2). Studies have been conducted, investigating multiple aspects of China’s healthcare system, including the reform, health insurance coverage and utilization, hospital management, cost, medication, and others (3–5).

China has a large aging population. It is projected that by 2050, the older population will make up a quarter of its total population. The middle-aged and elderly face fast deteriorating health conditions and increasing medical cost, and their health care is of special importance. When facing illness, a person may choose from inpatient treatment, outpatient treatment, and self-treatment. Inpatient treatment is defined as an appointment, procedure, and/or treatment requiring an overnight stay in a health facility. It usually corresponds to more severe illness conditions and poses a more serious health and financial threat.

Health cost has been an important topic and been studied in China as well as in many other countries (6–10). In previous studies, many efforts have been contributed to investigate medical costs and the associated factors for middle-aged and elderly people in China. Jiang and others studied the access to health care and the medical expenditure for the subjects aged 45 years and above who were randomly selected in mainland China (5). Wang and others investigated characteristics associated with insurance utilization and the association of treatment cost and insurance utilization for the middle-aged and elderly people with samples randomly collected in mainland China (11). For medical expenditure, associated factors that have been identified include demographic characteristics, such as age, gender, education and occupation, insurance status, living area, and so on (12–16). However, all the above studies do not have enough evidence to quantify the results specifically for middle-aged and elderly population who have inpatient treatment. Also, characteristics and effects of inpatient treatment in Central China have received less attention.

The goal of this study is to provide an updated and comprehensive description of inpatient treatment for the middle-aged and elderly (45 years old and above) in Central China. China is a large country with significant spatial variations. Of special interest is the highly populated, less-developed, and agriculture-dominating Central China. This area has a lower socioeconomic status and lower quality of health care, and as a result, illness may have more serious consequences. This study complements the existing literature along the following aspects. First, it focuses on inpatient treatment, which has characteristics significantly different from the other types of treatments. As inpatient treatment corresponds to more serious illness, it is more important from a healthcare perspective. Second, it comprehensively characterizes multiple aspects of inpatient treatment, including prevalence, characteristics, and cost. In contrast, most of the existing studies have focused on only a single aspect. Third, it focuses on the middle-aged and elderly, whose health care deserve more attention. Fourth, unlike many published studies, it is not limited to the treatment of a specific type of illness. Fifth, most of the existing studies were based on databases constructed by the central and local governments or hospitals. Such databases are designed to describe inpatient treatment from the healthcare providers’ perspective. Instead, in this study, data were collected using survey and can better describe inpatient treatment from the patients’ perspective. In addition, the survey also collected data on subjects without inpatient treatment for comparison. Such data are not available in hospital databases. Last, quite a few studies analyzed data collected on or before 2008. The reform launched in 2009 has fundamentally changed the healthcare system, and there is a strong need for an updated data collection and analysis.

## Materials and Methods

### Data Collection

The survey was conducted in August 2013. Samples were collected in the Henan province, which is the largest province in and representative of Central China. It is one of the least developed areas in China, highly populated, and agriculture-dominating. The study was approved by the Research Ethics Review Committee at the Renmin University of China.

At the beginning of each survey, the interviewer introduced the nature of the survey. Each interviewee was asked to sign an informed consent form. Information was collected to determine inclusion. An interviewee was excluded if he/she refused to participate, was younger than 45 years, or could not provide reliable information on illness conditions and expenditure. A total of 1,464 subjects finished the survey, with a response rate of 68%. The main reasons for refusal included “not comfortable with disclosing certain information,” “no time to finish survey,” and “concerns over how the results will be presented.” For over 95% of the non-responders, basic demographic information was collected. Analysis suggested comparability of the responders and non-responders.

The survey collected demographic and personal information, including gender, age, marital status, education, occupation, area (rural or urban), physical condition, income, presence of chronic disease, and health insurance coverage and utilization. To measure the accessibility of health care, information was collected on the distance to the nearest hospital and its type (17). Data were collected on all episodes of inpatient treatment during a period of 12 months prior to the survey. For each episode, data were collected on (a) illness leading to treatment, (b) distance to the hospital for treatment (which may differ from the nearest one) and its type, (c) reason(s) for choosing the specific hospital, (d) days of hospitalization, (e) treatment outcome, (f) cost (which includes cost of treatment, transportation/food/accommodation, medicine/supplies, unofficial gift, and lost income. The amount of insurance reimbursement, if insurance was utilized, was also collected.), (g) sources used to finance the cost (income, savings, funds from relatives and friends, and other sources), and (h) whether insurance was utilized. Cost was denominated in the unit RMB (100EUR = 819RMB by the exchange rate on 1st August 2013).

### Data Analysis

The subjects’ characteristics for the whole cohort and subgroups stratified by the number of inpatient treatments were summarized. Subjects with different inpatient treatment status were compared. *p* Values were computed from chi-squared tests for categorical variables and *t* tests for continuous variables. Summary characteristics of inpatient treatment were described for all episodes combined and for the first to sixth episodes of each subject. Multivariate analyses were conducted, controlling for confounders. The first set of multivariate analysis is on the frequency of inpatient treatment. Of special interest is the contrast between those with at least two inpatient treatments and those with only one. Logistic regression analysis was conducted, and the adjusted odds ratios (aOR) and *p*-values were conducted. The second set of analysis is on the pursuit of health care. Specifically, we analyzed whether grade III hospitals were used for treatment. The dominating majority of hospitals in China are public and under a unified grading system, with grade III hospitals providing the best quality of care and also being the most expensive (18). A binary variable was created to indicate whether a grade III hospital was used for treatment. Logistic regression analysis was conducted, and the odds ratios and their significance levels were computed. The third set of analysis is on the cost of inpatient treatment (for those with at least one episode). The first type of cost is the total cost, defined as the sum of cost on treatment, transportation/food/accommodation, medicine/supplies, unofficial gifts (to doctors and nurses), and lost income (due to illness). The second is the out-of-pocket (OOP) cost defined as the total cost minus insurance reimbursement. Linear regression was conducted. The estimated regression coefficients and their significance level were computed. Analysis was conducted using S-Plus Version 8.2 (TIBCO Software Inc.).

## Results

### Sample Characteristics

The results are shown in Table 1. A total of 582 subjects had at least one inpatient treatment, and 160 had more than one. In the comparison of patients’ characteristics of those with and without inpatient treatment, the age distribution is significantly different (*p* < 0.001), with those having inpatient treatments being older. The distribution of education is also significantly different (*p* = 0.007). For example, 20.7% in the group of no inpatient treatment had senior high school, compared to 15.3% in the group having inpatient treatment. Another significant variable is occupation (*p* = 0.009). Those with inpatient treatments are more likely to be from urban (59.6% versus 50.3%, *p* < 0.001) and have insurance (99.3% versus 97.7%, *p* = 0.02). In addition, they are more likely to have bad physical conditions (*p* < 0.001) and chronic diseases (*p* < 0.001). In the comparison of those with two or more inpatient treatments against those with one, age, physical condition, and presence of chronic disease are significant. The observed patterns are similar to those in the first set of comparison.

**Table 1:** **Characteristics for the whole cohort and the subgroups stratified by the number of inpatient treatment**.

	Total (*n* = 1,464)	Inpatient treatment = 0 (*n* = 882)	Inpatient treatment > 0 (*n* = 582)	*p*	Inpatient treatment = 1 (*n* = 422)	Inpatient treatment ≥ 2 (*n* = 160)	*p*
**Gender**				0.081			0.088
Male	626 (42.8)	361 (40.9)	265 (45.5)		183(43.4)	82 (51.3)	
Female	838 (57.2)	521 (59.1)	317 (54.5)		239 (56.6)	78 (48.8)	

**Age**	62.4 ± 10.7	61.0 ± 10.5	64.5 ± 10.6	<0.001	63.3 ± 10.5	67.7 ± 10.4	<0.001

**Age group**				<0.001			<0.001
45–50	254 (17.3)	181 (20.5)	73 (12.5)		64(15.2)	9 (5.6)	
51–60	423 (28.9)	284 (32.2)	139 (23.9)		103 (24.4)	36 (22.5)	
61–70	452 (30.9)	255 (28.9)	197 (33.8)		148 (35.1)	49 (30.6)	
>70	335 (22.9)	162 (18.4)	173 (29.7)		107 (25.4)	66 (41.3)	

**Marital status**				0.175			0.806
Single/divorced/widowed	250 (17.1)	141 (16.0)	109 (18.7)		78 (18.5)	31 (19.4)	
Married	1,213 (82.9)	740 (84.0)	473 (81.3)		344 (81.5)	129 (80.6)	

**Education**				0.007			0.455
No schooling	235 (16.2)	143 (16.3)	92 (15.9)		66 (15.8)	26 (16.4)	
Primary	411 (28.2)	220 (25.1)	191 (33.1)		130 (31.1)	61 (38.4)	
Junior high	438 (30.1)	269 (30.6)	169 (29.3)		130 (31.1)	39 (24.5)	
Senior high	270 (18.6)	182 (20.7)	88 (15.3)		65(15.6)	23 (14.5)	
Junior college and above	101 (6.9)	64 (7.3)	37 (6.4)		27 (6.5)	10 (6.3)	

**Occupation**				0.009			0.109
Governments	74 (5.1)	49 (5.6)	25 (4.3)		19 (4.5)	6 (3.8)	
Enterprises	65 (4.4)	38 (4.3)	27 (4.6)		14 (3.3)	13 (8.1)	
Farmers	551 (37.6)	351 (39.8)	200 (34.4)		154 (36.5)	46 (28.8)	
Small private business	53 (3.6)	38 (4.3)	15 (2.6)		13 (3.1)	2 (1.3)	
Other^a^	47 (3.2)	34 (3.9)	13 (2.2)		10 (2.4)	3 (1.9)	
Retired	450 (30.7)	245 (27.8)	205 (35.2)		144 (34.1)	61 (38.1)	
No jobs	224 (15.3)	127 (14.4)	97 (16.7)		68 (16.1)	29 (18.1)	

**Area**				<0.001			0.150
Urban	791 (54.0)	444 (50.3)	347 (59.6)		244(57.8)	103 (64.4)	
Rural	673 (46.0)	438 (49.7)	235 (40.4)		178 (42.2)	57 (35.6)	

**Health insurance coverage**				0.020			
Yes	1,440 (98.4)	862 (97.7)	578 (99.3)		418 (99.1)	160 (100.0)	
No	24 (1.6)	20 (2.3)	4 (0.7)		4 (0.9)	0	

**Distance to the nearest hospital (meter)**				0.068			0.879
≤500	1,061 (72.5)	658 (74.6)	403 (69.2)		294 (69.7)	109 (68.1)	
501–1,000	236 (16.1)	134 (15.2)	102 (17.5)		74 (17.5)	28 (17.5)	
≥1,001	167 (11.4)	90 (10.2)	77 (13.2)		54 (12.8)	23 (14.4)	

**Type of the nearest hospital**				0.065			0.248
Grade I	867 (59.3)	539 (61.2)	328 (56.4)		248 (58.8)	80 (50.0)	
Grade II	413(28.2)	237 (26.9)	176 (30.2)		119(28.2)	57 (35.6)	
Grade III	88 (6.0)	44 (5.0)	44 (7.6)		30 (7.1)	14 (8.8)	
Private	95 (6.5)	61 (6.9)	34 (5.8)		25 (5.9)	9 (5.6)	

**Average personal income (1K RMB)**	12.8 ± 23.9	12.4 ± 28.3	13.4 ± 15.0	0.435	13.3 ± 15.3	13.7 ± 14.3	0.796

**Physical condition**				<0.001			<0.001
Healthy	346 (23.6)	290 (32.9)	56 (9.6)		48 (11.4)	8 (5.0)	
Just so-so	655 (44.7)	416 (47.2)	239 (41.1)		191(45.3)	48 (30.0)	
A little sick	258 (17.6)	113 (12.8)	145 (24.9)		102 (24.2)	43 (26.9)	
Sick	168 (11.5)	56 (6.3)	112 (19.2)		69 (16.4)	43 (26.9)	
Very sick	37 (2.5)	7 (0.8)	30 (5.2)		12 (2.8)	18 (11.3)	

**Chronic disease**				<0.001			0.005
No	241 (16.5)	203 (23.0)	38 (6.5)		35 (8.3)	3 (1.9)	
Yes	1,223 (83.5)	679 (77.0)	544 (93.5)		387 (91.7)	157 (98.1)	

**Number of inpatient treatment**	–	–	1.4 ± 0.8		1.0 ± 0.0	2.5 ± 0.9	

*For a categorical variable, count (percentage). For a continuous variable, mean ± SD*.

*^a^“Other” means the other occupations except governments, enterprises, farmers, and small private business*.

### Characteristics of Inpatient Treatment Episodes

The 582 subjects had a total of 823 episodes of inpatient treatments (Table 2). The numbers of subjects with 1–6 episodes are 582, 160, 51, 16, 10, and 4, respectively. With small counts, statistics for the fourth to sixth episodes are less reliable. 98.4% of the episodes were treated in public hospitals with the majority of which being in grade II (45.7%) and III (40.4%) hospitals. The distributions across the first three episodes are similar. The average distance to hospital is about 56 km. Multiple factors contributed to the choice of hospital, with the most common concern being the quality of treatment (58.8%), followed by easy-to-use insurance (38.0%) and close distance (36.0%). The average days of hospitalization is 17.8, and a decreasing trend across treatments is observed (18.5, 16.7, and 15.2 days for the first to third treatments). The outcomes are mostly positive, with 14.0% cured and 79.8% getting better. The distributions differ across treatments. For example, 16.3%, 10.7%, and 5.9% were cured for the first to third episodes, respectively. The average gross total cost is about 14,478RMB. The largest cost category is treatment, followed by lost income, transportation/food/accommodation, and medicine/supplies. The average insurance payment is 5,521RMB, and the average OOP cost is 9,260RMB. In terms of financing, 65.2% was funded by income, followed by funds from relatives and friends (20.6%) and savings (11.4%). Differences are observed across episodes. Insurance was used for the majority of the episodes (93.6%).

**Table 2:** **Description of inpatient treatment episodes, for all episodes combined and the first to sixth episodes of each subject**.

	Total (*n* = 823)	Order of inpatient treatment episode					
		First (*n* = 582)	Second (*n* = 160)	Third (*n* = 51)	Fourth (*n* = 16)	Fifth (*n* = 10)	Sixth (*n* = 4)
**Type of hospital**							
Grade I	101 (12.3)	74 (12.8)	23 (14.4)	4 (7.8)	0	0	0
Grade II	375 (45.7)	266 (45.9)	75 (46.9)	21(41.2)	9 (56.3)	3 (30.0)	1 (25.0)
Grade III	331 (40.4)	230 (39.7)	61(38.1)	24 (47.1)	6 (37.5)	7 (70.0)	3 (75.0)
Private	13 (1.6)	9 (1.6)	1 (0.6)	2 (3.9)	1 (6.3)	0	0
Distance to hospital (m)	56,166.6 ±437,444.1	63,512.2 ±475,744.5	48,212.2 ±398,863.5	21,578.7 ±72,076.0	4,578.1 ±10,381.7	36,875.0 ±93,347.4	1,125.0 ±250.0

**Reason(s) for choosing the specific hospital**							
Close distance	296(36.0)	197 (33.8)	59 (36.9)	20 (39.2)	13 (81.3)	5 (50.0)	2 (50.0)
Better treatment	484 (58.8)	345 (59.3)	94 (58.8)	31 (60.8)	5 (31.3)	6 (60.0)	3 (75.0)
Easy-to-use insurance	313 (38.0)	219 (37.6)	62 (38.8)	16 (31.4)	8 (50.0)	5 (50.0)	3 (75.0)
Easy-to-get appointment	31 (3.8)	19 (3.3)	5 (3.1)	3 (5.9)	2 (12.5)	1 (10.0)	1 (25.0)
Other	67 (8.1)	54 (9.3)	9 (5.6)	3 (5.9)	0	1 (10.0)	0
Days of hospitalization	17.8 ± 20.2	18.5 ± 22.2	16.7 ± 16.9	15.2 ± 6.8	14.7 ± 9.9	14.9 ± 8.8	15.5 ± 10.2

**Treatment outcome**							
Cured	115 (14.0)	95 (16.3)	17 (10.7)	3 (5.9)	0	0	0
Better	656 (79.8)	455 (78.2)	129 (81.1)	43 (84.3)	15 (93.8)	10 (100.0)	4 (100.0)
Same	45 (5.5)	28 (4.8)	12 (7.5)	4 (7.8)	1 (6.3)	0	0
Worse	6 (0.7)	4 (0.7)	1 (0.6)	1 (2.0)	0	0	0

**Cost (RMB)**							
Treatment	10,971.2 ± 22,882.2	11,862.3 ± 25,886.5	8,597.6 ± 11,619.3	7,048.4 ± 6,293.0	9,618.8 ± 14,093.9	20,300.0 ± 35,799.8	8,375.0 ± 2,868.7
Transportation/food/accommodation	728.1 ± 1,842.1	788.3 ± 2,080.9	528.4 ± 1,039.6	580.4 ± 702.0	656.3 ± 843.8	1,350.0 ± 2,392.2	575.0 ± 613.1
Medicine/supplies	409.4 ± 1,766.9	483.9 ± 2,054.2	228.4 ± 675.7	269.6 ± 742.8	187.5 ± 338.4	100.0 ± 253.9	250.0 ± 500.0
Unofficial gift	61.0 ± 625.1	75.7 ± 738.6	25.7 ± 133.8	9.8 ± 70.0	31.3 ± 125.0	50.0 ± 158.1	125.0 ± 250.0
Lost income	932.9 ± 3,558.1	966.8 ± 3,724.1	618.6 ± 1,515.3	658.0 ± 1,754.6	2,473.1 ± 8,979.8	3,067.0 ± 7,551.1	587.5 ± 956.0
Gross total cost	14,477.7 ± 30,194.0	15,100.1 ± 29,100.9	10,827.3 ± 14,448.0	8,990.4 ± 7,726.2	12,929.4 ± 22,738.4	24,667.0 ± 45,215.9	120,621.3 ± 219,852.5
Paid by insurance	5,521.5 ± 9,702.7	6,325.8 ± 11,151.8	4,862.1 ± 6,051.5	4,236.6 ± 3,674.4	5,761.5 ± 5,369.4	7,555.6 ± 8,453.7	5,500.0 ± 3,968.6
Out-of-pocket cost	9,260.1 ± 26,714.6	9,495.5 ± 23,856.3	6,609.2 ± 11,213.5	4,950.2 ± 5,285.5	9,290.0 ± 20,358.3	18,818.9 ± 39,848.6	152,661.7 ± 255,703.0

**Financial sources**							
Income	65.2%	67.7%	62.1%	55.8%	48.8%	53.0%	30.0%
Savings	11.4%	11.6%	11.3%	11.0%	6.9%	10.0%	20.0%
Funds from relatives/friends	20.6%	17.2%	24.9%	32.3%	44.4%	37.0%	50.0%
Other	2.9 ± 13.8%	3.5 ± 15.5%	1.8 ± 9.0%	1.0 ± 7.0%	0	0	0

**Insurance utilization**							
Yes	770 (93.6)	545 (93.6)	150 (93.8)	46 (90.2)	15 (93.8)	10 (100.0)	4 (100.0)
No	53 (6.4)	37 (6.4)	10 (6.3)	5 (9.8)	1 (6.3)	0	0

*For a categorical variable, count (percentage). For a continuous variable, mean ± SD, i.e., confident interval*.

Multiple illness conditions led to inpatient treatment. The most common is cardiovascular and cerebrovascular diseases (36.8%), followed by stomach and digestive diseases (10.9%), hypertension (7.7%), and others. Many other conditions, such as cervical spine diseases and trauma fractures, also led to inpatient treatments, but are less common.

### Frequency of Inpatient Treatment

In analysis, 32 records with missing measurements are removed, leading to an effective sample size of 550. The results are shown in Table 3. The association for age is significant. With the 45- to 50-year group as reference, the 51–60, 61–70, and 70+ age groups have aORs 2.472, 2.517, and 4.46, respectively. In addition, the presence of chronic disease is significant, with an aOR 4.229 (*p* = 0.024).

**Table 3:** **Multivariate logistic regression analysis of the number of inpatient treatments: at least two times versus once (reference)**.

	aOR (95% CI)	*p*
**Gender (reference: male)**		
Female	0.829 (0.537–1.281)	0.399

**Age group (reference: 45–50)**		
51–60	2.472 (1.049–5.827)	0.039
61–70	2.517 (1.067–5.939)	0.035
>70	4.460 (1.805–11.017)	0.001

**Marital status (reference: single/divorced/widowed)**		
Married	0.980 (0.568–1.689)	0.941

**Education (reference: no schooling)**		
Primary	1.124 (0.612–2.062)	0.707
Junior high	0.926 (0.476–1.800)	0.820
Senior high	0.915 (0.423–1.980)	0.822
Junior college and more	1.065 (0.379–2.991)	0.905

**Occupation (reference: governments)**		
Enterprises	2.706 (0.732–10.009)	0.136
Farmers	0.674 (0.193–2.353)	0.536
Small private business	0.522 (0.080–3.414)	0.498
Others	0.960 (0.176–5.243)	0.962
Retired	0.912 (0.309–2.688)	0.867
Unemployed	0.949 (0.283–3.186)	0.933

**Area (reference: urban)**		
Rural	1.088 (0.579–2.044)	0.794

**Chronic disease (reference: no)**		
Yes	4.229 (1.212–14.758)	0.024

**Insurance utilization (reference: yes)**		
No	1.852 (0.806–4.256)	0.146
Per capita income (1K RMB)	0.998 (0.983–1.013)	0.762

*n = 550 (after removing records with missing measurements)*.

*aOR, adjusted odds ratio*.

### Utilization of Grade III Hospitals

Different treatment episodes have different characteristics (Table 2). In this analysis, we focus on the first episodes of all subjects. Analysis on the rest five episodes is not conducted with a sample size consideration. Three records with missing measurements are removed, leading to an effective sample size of 579. The analysis results are shown in Table 4. Those being female (aOR 1.594, *p* = 0.024), being married (aOR 2.266, *p* = 0.004), and having a higher income (aOR 1.014, *p* = 0.047) are more likely to use grade III hospitals.

**Table 4:** **Analysis on the utilization of grade III hospitals for inpatient treatment**.

	Using grade III hospital for inpatient treatments (*n* = 579)			
	Total	Using grade III hospital, *n* (%)	OR (*p*)	aOR (*p*)
**Gender**				
Male	264	99 (37.5)	–	–
Female	315	131 (41.6)	1.187 (0.317)	1.594 (0.024)

**Age**				
45–50	72	23 (31.9)	–	–
51–60	137	53 (38.7)	1.344 (0.336)	0.998 (0.996)
61–70	197	81 (41.1)	1.488 (0.173)	1.145 (0.689)
>70	173	73 (42.2)	1.555 (0.136)	1.076 (0.846)

**Marital status**				
Single/divorced/widowed	109	34 (31.2)	–	–
Married	470	196 (41.7)	1.578 (0.044)	2.266 (0.004)

**Education**				
No schooling	92	28 (30.4)	–	–
Primary	190	77 (40.5)	1.558 (0.101)	1.452 (0.225)
Junior high	167	66 (39.5)	1.494 (0.147)	1.030 (0.928)
Senior high	88	38 (43.2)	1.737 (0.077)	1.059 (0.880)
Junior college and more	37	19 (51.4)	2.413 (0.027)	1.097 (0.852)

**Occupation**				
Governments	25	10 (40.0)	–	–
Enterprises	27	10 (37.0)	0.882 (0.826)	1.130 (0.843)
Farmers	198	57 (28.8)	0.606 (0.253)	0.608 (0.396)
Small private business	15	5 (33.3)	0.750 (0.674)	0.648 (0.564)
Others	13	5 (38.5)	0.938 (0.927)	1.339 (0.704)
Retired	205	112 (54.6)	1.806 (0.171)	1.684 (0.297)
Unemployed	96	31 (32.3)	0.715 (0.469)	0.734 (0.585)

**Area**				
Urban	346	165 (47.7)	–	–
Rural	233	65 (27.9)	0.424 (< 0.001)	0.806 (0.478)

**Chronic disease**				
No	38	13 (34.2)	–	–
Yes	541	217 (40.1)	1.288 (0.473)	1.211 (0.629)

**Insurance utilization**				
Yes	542	217 (40.0)	–	–
No	37	13 (35.1)	0.811 (0.556)	0.804 (0.597)
Per capita income (1K RMB)	–	–	1.026 (<0.001)	1.014 (0.047)

*OR, odds ratio from univariate logistic regression*.

*aOR, adjusted odds ratio from multivariate logistic regression*.

### Cost of Treatment

The multivariate analysis results are presented in Table 5. As above, analysis is conducted on the first episodes of all subjects. Removing records with missing measurements leads to effective sample sizes of 548 and 508, respectively, for total and OOP cost. In the analysis of total cost, education is significant. With no schooling as the baseline, those with senior high and junior college and more spent 11.9 K RMB (*p* = 0.018) and 13.6 K RMB (*p* = 0.048) more, respectively. The type of hospital used for treatment is also significant. With grade I hospital as the baseline, subjects using grade III hospital spent 12.6 K RMB more (*p* = 0.003). Another significant variable is insurance utilization. Not using insurance led to 12.3 K RMB more cost (*p* = 0.024). In the analysis of OOP cost, the same set of variables is found to be significant. For education, only the senior high category is significant, with an estimated coefficient of 10.6 K (*p* = 0.014). Using grade III hospital leads to 9 K more cost than using grade I hospital (*p* < 0.001). Not using insurance leads to 17.4 K more cost (*p* < 0.001).

**Table 5:** **Multivariate regression analysis of total and out-of-pocket (OOP) cost**.

	Total cost (*n* = 548)	OOP cost (*n* = 508)
Gender (reference: male)	−1,623.4 (0.561)	−649.1 (0.785)

**Age group (reference: 45–50)**		
51–60	2,947.2 (0.520)	1,012.6 (0.792)
61–70	4,130.0 (0.359)	1,319.2 (0.728)
>70	2,771.7 (0.581)	382.4 (0.928)

**Marital status (reference: single/divorced/widowed)**		
Married	673.5 (0.853)	607.1 (0.842)

**Education (reference: no schooling)**		
Primary	−359.8 (0.929)	−66.9 (0.984)
Junior high	915.5 (0.831)	537.6 (0.883)
Senior high	11,943.0 (0.018)	10,566.4 (0.014)
Junior college and more	13,637.7 (0.048)	6,580.8 (0.274)

**Occupation (reference: governments)**		
Enterprises	−2,472.8 (0.775)	−1,224.7 (0.867)
Farmers	10,374.9 (0.194)	9,687.1 (0.147)
Small private business	10,299.4 (0.315)	9,689.9 (0.262)
Others	−5,571.3 (0.606)	−6,442.7 (0.481)
Retired	2,625.7 (0.705)	2,880.3 (0.617)
No jobs	8,889.8 (0.253)	9,169.1 (0.157)

**Areas (reference: urban)**		
Rural	−7,019.0 (0.086)	−5,970.1 (0.088)

**Type of hospital (reference: grade I)**		
Grade II	117.5 (0.976)	559.7 (0.867)
Grade III	12,555.3 (0.003)	9,002.9 (0.010)
Private	−6,442.9 (0.564)	−8,461.6 (0.416)

**Chronic disease (reference: no)**		
Yes	1,022.3 (0.846)	913.0 (0.835)

**Insurance utilization (reference: yes)**		
No	12,346.0 (0.024)	17,375.1 (<0.001)
Per capita income (1K RMB)	−64.0 (0.481)	−57.8 (0.443)

*In each cell, estimated regression coefficient (*p*-value)*.

## Discussion

China has a fast aging population. Illness conditions, treatment, and their consequences for the middle-aged and elderly are of significant interest for healthcare providers, researchers, and policy makers. This is especially true for the less-advantaged areas, for example, the highly populated and agriculture-dominating Central China as surveyed in this study.

Using survey, this study is able to better characterize several important aspects of inpatient treatment. Specifically, studies based on hospital databases do not have information on people without treatment, and they are likely to be biased due to the specialty, type, and location of hospitals. In comparison, this study better describes inpatient treatment on the population level. The prevalence of inpatient treatment is found to be high, with 39.8% of the surveyed subjects having at least one episode. Illness that leads to inpatient treatment has serious health and financial consequences (19). The observed high prevalence deserves special attention. The illness conditions that led to inpatient treatments are highly correlated with aging. The frequency of different illness observed in this study can assist better distributing healthcare resources.

Inpatient treatments dominatingly happened in grade II and III hospitals. With an average travel distance of 56 km and a high variation, the accessibility to health care is less satisfactory, at least for some subjects. Similar concerns have been raised in the literature (20). Treatment quality was of the most concern for choosing a specific hospital, which is reasonable considering the special nature of inpatient treatment. Accessibility and insurance utilization also play important roles. Under an effective healthcare system, such factors should play minimal roles in healthcare pursuit. Further work is needed to improve accessibility and to reduce the obstacles in using insurance (11, 17). The treatment results are dominatingly positive, with only 5.5% staying the same and 0.7% getting worse. Inpatient treatment is expensive (21, 22). The average gross total and OOP costs are 14.5 K and 9.3 K RMB, respectively. In the Henan province, the per capita GDP is 24.7 K RMB. Even though insurance covers a significant amount of cost (5.5 K on average), the remaining OOP cost is still high and can pose a serious financial burden, which is consistent with the study comparing health expenditure spending with the economic growth in Brazil, Russia, India, China, and South Africa (8). Cost for the majority of episodes was funded by income and savings. However, cost of 20.6% of the episodes had to be supported by funds from relatives and friends, which causes a long-term financial burden. The healthcare and insurance systems need improvement to alleviate the financial consequences (13). The insurance utilization rate is high, which is different from that reported in some recent studies (11). The subjects had up to six episodes of inpatient treatments, and different episodes had different characteristics. The variations across episodes are associated with multiple factors, especially the characteristics of illness.

Only age and the presence of chronic disease are associated with the frequency of treatment. The positive association for age is reasonable, as the common illness conditions leading to treatment are aging-related. The presence of chronic disease has been adopted as a surrogate for overall health conditions (11, 17, 20), and the observed positive association is consistent with the literature. Other personal and demographic characteristics are not associated with frequency. This observation differs from that in some recent studies (14, 17). The result is “positive” in the sense that no less-advantaged group is identified. The difference may be partly attributable to the homogeneity of survey subjects.

Multiple factors contribute to the utilization of grade III hospitals for inpatient treatment. Gender, marital status, and education are found significant. The pursuit of health care is a complex process (23, 24). These factors may contribute via a psychological way as well as associate with other factors. It is noted that in marginal analysis, subjects in rural areas are less likely to use grade III hospital (24); in univariate and multivariate analyses, per capita income is significant. Such results may suggest disparity. Grade III hospitals provide the highest quality of care. In this sense, those living in rural and/or with lower income are less advantaged. Meanwhile, the higher inpatient treatment copayments are required for the rural citizens, so they are more likely to drop out of the inpatient treatment than citizens in urban areas (25). The inequalities of health resources and thus treatment gaps in rural and urban areas have also been investigated in China, India, and U.S. in other studies (26, 27). Further adjustment of the healthcare and insurance system is needed to eliminate disparity (25).

Health expenditure has been an important topic and been studied in China as well as in many other countries (6–10). In the analysis of cost, education, type of hospital, and insurance utilization are significant. The significant association for education is partly confounded by the type of hospital used. The high cost of grade III hospitals is associated with the high quality of care. It may also be correlated with the severity of illness. Unfortunately, such information cannot be obtained from survey. Medical records from hospitals have to be collected and analyzed. A small number of subjects did not use insurance in their inpatient treatments. Under the present system, insurance utilization is not automatic, and recent studies have found that there are still a small number of patients who had but did not use insurance (11). The survey did not collect information on why insurance was not used, and thus, further analysis is not conducted (17). However, in spite of the high insurance utilization in inpatient treatment in our study, it may not implicate satisfactory outcomes (28), and thus calls for more attention in the further study.

This study has limitations. To collect certain specific information (for example, the prevalence of inpatient treatment, amount of lost income, etc.), survey was used. Survey data have limitations, including possible recall bias and limited information (29). More detailed information on illness, treatment, and outcome has to be obtained from hospitals. As the surveyed subjects used quite a few different hospitals, collecting hospital data is not feasible. When selecting samples, we strived to achieve randomness and representativeness. We have examined data in multiple ways, and there is no obvious sign of sampling bias. However, without having access to more detailed data on the surveyed areas, we are not able to fully confirm representativeness. With limited resources, sample collection was limited to the Henan province, which is representative of Central China. Health conditions and health care vary significantly in China (30). A counterpart study in other areas is also of interest.

## Conclusion

This study has provided an updated and comprehensive description of inpatient treatment for the middle-aged and elderly in Central China. It has been found that inpatient treatment has a high prevalence. Its characteristics, age, and the presence of chronic disease are significantly associated with the number of inpatient treatments. The findings can assist healthcare providers to better reform the system. Multiple factors are identified as associated with grade III hospital utilization and cost. Policy interventions are needed to make hospitals more accessible and more affordable.

## Author Contributions

All the authors contributed to the study design. YJ, YW, YL, YZ, and XW conducted the survey and data collection. YW and YZ conducted statistical analysis. YZ, CM, and SM drafted the manuscript. The final manuscript was read and approved by all authors.

## Conflict of Interest Statement

The authors declare that the research was conducted in the absence of any commercial or financial relationships that could be construed as a potential conflict of interest.

## Abbreviations

OOP, out-of-pocket; aOR, adjusted odds ratio.
